# Orofacial Muscle Weakening in Facioscapulohumeral Muscular Dystrophy (FSHD) Patients

**DOI:** 10.3390/children9010096

**Published:** 2022-01-11

**Authors:** Dimitrios Konstantonis, Kyriaki Kekou, Petros Papaefthymiou, Heleni Vastardis, Nikoleta Konstantoni, Maria Athanasiou, Maria Svingou, Anastasia Margariti, Angeliki Panousopoulou

**Affiliations:** 1 Department of Orthodontics, School of Dentistry, National and Kapodistrian University of Athens, GR-115 27 Athens, Greece; h.vastardis@gmail.com; 2 Clinic of Orthodontics and Pediatric Dentistry, Center of Dental Medicine, University of Zurich, CH-8032 Zurich, Switzerland; 3 Laboratory of Medical Genetics, Children’s Hospital Agia Sophia, National and Kapodistrian University of Athens, GR-115 27 Athens, Greece; kkekou@med.uoa.gr (K.K.); m.sviggou@yahoo.gr (M.S.); 4 Department of Orthodontics, Faculty of Dentistry, Marmara University, Istanbul 34854, Turkey; papaefthimiou.petros4@gmail.com; 5 ATX Braces & Smile Aligners, In Private Practice of Orthodontics, 1601 E Pflugerville Pkwy Building 2, Pflugerville, TX 78660, USA; nikonstantoni@gmail.com; 6 Athensbestsmiles, In Private Practice of Orthodontics, 49 Alopekis, GR-106 76 Athens, Greece; athanasioumaria994@gmail.com; 7 Department of Elderly Medicine, University Hospital Sussex NHS Foundation Trust, St. Richard’s Hospital, Spitalfield Ln, Chichester PO19 6SE, UK; anastamarg@gmail.com; 8 Department of Neurology, Korgialenio-Benakio Hellenic Red Cross Hospital, GR-115 26 Athens, Greece; agpanousopoulou@gmail.com

**Keywords:** muscle weakness, muscular dystrophy, D4Z4, genotype-phenotype correlation, genetic testing

## Abstract

Background: Facioscapulohumeral muscular dystrophy is the third most commonly found type of muscular dystrophy. The aim of this study was to correlate the D4Z4 repeat array fragment size to the orofacial muscle weakening exhibited in a group of patients with a genetically supported diagnosis of FSHD. Methods: Molecular genetic analysis was performed for 52 patients (27 female and 25 male) from a group that consisted of 36 patients with autosomal dominant pedigrees and 16 patients with either sporadic or unknown family status. The patients were tested with the southern blotting technique, using EcoRI/Avrll double digestion, and fragments were detected by a p13E-11 telomeric probe. Spearman’s correlation was used to compare the fragment size with the degree of muscle weakening found in the forehead, periocular and perioral muscles. Results: A positive non-significant correlation between the DNA fragment size and severity of muscle weakness was found for the forehead (r = 0.27; *p* = 0187), the periocular (r = 0.24; *p* = 0.232) and the left and right perioral (r = 0.29; *p* = 0.122), (r = 0.32; *p* = 0.085) muscles. Conclusions: Although FSHD patients exhibited a decrease in muscular activity related to the forehead, perioral, and periocular muscles the genotype–phenotype associations confirmed a weak to moderate non-significant correlation between repeat size and the severity of muscle weakness. Orofacial muscle weakening and its association with a D4Z4 contraction alone may not have the significance to serve as a prognostic biomarker, due to the weak to moderate association. Further studies with larger sample sizes are needed to determine the degree of genetic involvement in the facial growth in FSHD patients.

## 1. Introduction

Facioscapulohumeral Muscular Dystrophy (FSHD, MIM#158900) is one of the most common types of muscular dystrophy, which is inherited in autosomal dominant pattern. FSHD has an estimated prevalence of 1:20,000, an insidious onset, and is characterized by the progressive weakness of the facial, shoulder girdle and foot extensor muscles [1].

FSHD is the third most common type of inherited muscle disease after Duchenne muscular dystrophy and myotonic dystrophy [2]. There are several estimations about the epidemiology of the FSHD with recent studies suggesting a prevalence of approximately 1 in 15,000 people. It is also suggested that the actual prevalence may differ (1:8300) due to the wide spectrum of disease severity and the fact that about 20% of genetically confirmed patients seem to be asymptomatic [3]. Moreover, according to recently conducted studies, large phenotypic variability is connected with heterozygote subjects carrying D4Z4 alleles of different sizes [4,5,6]. FSHD has a variable age of onset that can occur from infancy to adult life along with a rather benign course and a strong penetrance.

Clinical hallmarks of FSHD are the regional distribution of weakness and the remarkable intra- and interfamilial variability, ranging from asymptomatic carriers to severe clinical phenotypes [7]. The name of the disorder comes from the muscles preferentially affected. Facial muscles, muscles that fixate the scapula, and muscles overlying the humerus are typically weakened. FSHD can be diagnosed at any age. There is also a wide range of onset and severity of FSHD cases found from asymptomatic carriers of the allele to rapidly progressive cases [8].

Patients often report problems in performing activities above their shoulders, such as the inability to whistle, hearing impairment, and a change in their physical appearance due to atrophy and muscle weakening while exhibiting scapular winging and a protuberant abdomen [9]. It is quite common for patients to experience pain and fatigue. The physical limitations are significant, resulting in either disability or some type of job modification. Of the affected individuals, 20% eventually require a wheelchair. However, they do have a normal life expectancy. Today, there are no disease-modifying treatments available beyond supportive care [10].

### 1.1. Genetics of FSHD

FSHD is inherited in an autosomal dominant pattern; however, approximately 10% of the cases are due to de novo mutations. There are two phenotypically identical types: FSHD1 (representing 95% of cases) and FSHD2 (5% of cases). The disease is caused by the toxic expression of the DUX4, a retrogene contained in every D4Z4 macrosatellite repeat array on chromosome 4q35, expressed in the germline but typically repressed in somatic tissue. The derepression of the DUX4 occurs from an opening of the chromatin structure either by contraction of the number of repeats (FSHD1) or by chromatin hypomethylation of the D4Z4 repeats stemming from mutations in SMCHD1, DNMT3B and LRIF1, genes involved in chromatin methylation (FSHD2) [11,12,13,14,15]. Individuals suffering from FSHD1 carry a reduced number of 1 to 10 D4Z4 repeats (each 3.3 kb), while non patients carry a number of 11 to 100 repeats. FSHD2 patients carry a normal number of repeats but typically fewer (11–20) compared to the control population [16].

Recent studies showed that the human retrogene DUX4 activates target genes such as PAX7 that are proposed to drive muscular dystrophy pathology in a toxic gain-of-function model [17]. Moreover, the FSHD2 subtype seems to arise through a common to FSHD1 downstream mechanism of epigenetic derepression including the epigenetic modifier of D4Z4 repression DNMT3B and LRIF1 [13].

### 1.2. Craniofacial Discrepancies in Muscular Dystrophies

Dentofacial irregularities are present in individuals without any syndromes or pathology [18]. One of the main factors which, influence the growth direction of the dentofacial skeleton are the forces generated by the facial musculature [19]. Experimental restrictions of muscle activity led to changes of bone shape and size. In numerous investigations conducted on animals, bisection of a specific muscle or septum attached to a bone leads to inadequate osseous development [20]. Clinical investigations have shown that the decrease of muscular tone in patients with myopathies or muscle weakening syndromes leads to severe disruption of jaw development. Common features of muscular disorders are the increased vertical dimension of the face, expressed in the maxillofacial complex by compensatory overeruption of the posterior teeth and subsequent severe anterior and lateral open bite. Additionally, deviations in the transverse dimension of the maxilla are frequently present [21,22].

According to the “equilibrium” theory the resultant of all forces exerted by the neuromuscular system as well as from the oral functions (breath, speech, mastication, deglutition etc.) must be in absolute equilibrium for the facial skeleton to develop normally and to provide tissue homeostasis. The “functional matrix” hypothesis suggests that skeletal development is adaptive or secondary to soft tissue growth of the soft tissue, organs and spaces of the craniofacial region [23].

In the literature, there is a lack of studies with regard to the dento- and craniofacial development as well as the orthodontic diagnosis and management of FSHD patients. However, some authors have investigated the effects of myotonic dystrophy and Duchenne muscular dystrophy on the orofacial muscles and dentofacial morphology. Additionally, a few clinical cases of combined orthodontic and surgical treatment have been reported [24,25].

It is therefore the aim of this study, to correlate the D4Z4 fragment size to the weakening of the facial mimic muscles triad in a patient group with genetically confirmed FSHD diagnosis.

## 2. Materials and Methods

**Figure 1:**
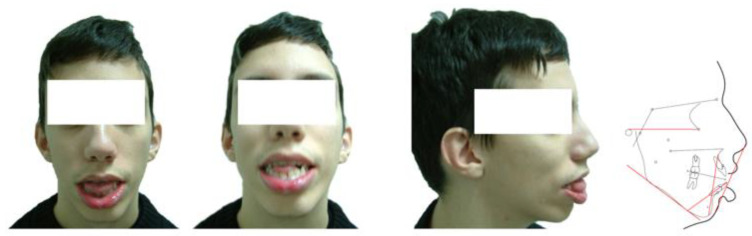
Patient P1: portrait photographs and cephalometric tracing of a male FSHD patient, 19 years old who presented with documented early onset of the disease and a genetic testing showing a substantial fragment. The patient was identified as a sporadic case. The clinical screening as well as the cephalometric analysis revealed a long ovoid face with increased anterior lower facial height, anterior open bite and posterior bilateral crossbite. The lips were severely incompetent and protrusive and the tongue was oversized.

Patients were recruited from the outpatient neuromuscular clinic of the Children’s Hospital “Agia Sophia”, which is a part of the National and Kapodistrian University of Athens, Greece. Patients with FSHD phenotype were examined by two neurologists, familiar with the diagnosis of FSHD.Σθβ. In the present study, we excluded patients with the typical facioscapulohumeral phenotype without FSHD 4q35 deletion as we considered them to have limb-girdle muscular dystrophy, facial sparing scapulohumeral dystrophy or classical FSHD2 phenotype without an EcoRI/BlnI-fragment smaller than 40 kb. The latter patients had either mild phenotype or borderline EcoRI/Avrll-fragments and this is in agreement with other researchers. After the completion of the clinical study 52 Caucasian patients, 25 male and 27 female, with a mean age of 41.83 (SD 17.29) years who fulfilled both the clinical and genetic criteria of FSHD as outlined by the European Neuromuscular Centre FSHD Consortium, were included in our study. In the absence of facial weakness, a diagnosis can only be accepted if the majority of family members exhibit facial weakness according to the diagnostic criteria [26]. Among the 52 patients, 36 were familial cases and 16 isolated ones were ascertained, the latter, being either sporadic (Figure 1) or with an unidentified family history. Familial cases included 14 autosomal dominant pedigrees that had two to six affected subjects each in two, three or four generations. The study protocol was approved by the Institutional Review Board of the Children’s Hospital “Agia Sophia” of the National and Kapodistrian University of Athens, Greece (24310/29 October 2015) and this study was conducted according to the principles of the Declaration of Helsinki (version October 2013). Informed consent for genetic analysis and photos was provided by all individuals tested (or their legal guardians).

For genetic analysis, high molecular DNA was extracted from peripheral blood lymphocytes and further analyzed by using the molecular diagnostic protocol according to the salting out method and the molecular procedure used by Kekou et al. [27]. In brief, 10 μg of genomic DNA were digested using EcoRI and AvrII restriction enzymes according to the manufacturer’s instructions (New England Biolabs Cat.Nos. R101L and R174L, respectively). Ιn order tο aνoid radioactiνe labelling, the probe p13E-l1 was labelled by DIG dUTP incorporation during a PCR procedure using a PCR DIG Probe Synthesis Κit (Roche, Cat. Νο. 1636090). Both conνentional and pulsed-field gel electrophoresis were performed as reported by den Dunnen et al. [28].

**Figure 2:**
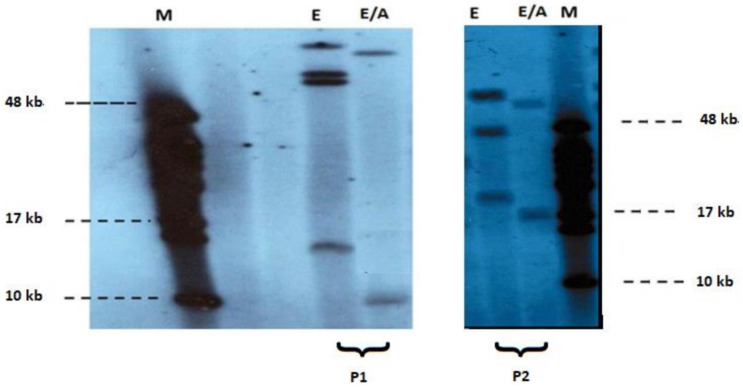
Pulsed-field gel electrophoresis-based FSHD diagnosis of EcoRI (1) and EcoRI/AvrII (2) digested DNA and subsequent non-radioactive Southern blot analysis using p13E-11 probe in patients P1, P2. Clinical diagnosis of FSHD was confirmed by the detection of short EcoRI/AvrII (E/A) fragments (~9 kb and 17 kb respectively) M: 48 kb marker (Biolabs).

At least one proband in each pedigree and all of the 16 apparently isolated cases underwent molecular analysis, with southern blotting technique, using EcoRI/AvrII double digestion, while fragments were detected by p13E-11 telomeric probe [gel] (Figure 2). Parents’ DNA was examined in only one of the isolated cases, while in the rest the genetic background remained obscure. Among the patients studied, seven sporadic cases—four males and three females—were identified.

**Figure 3:**
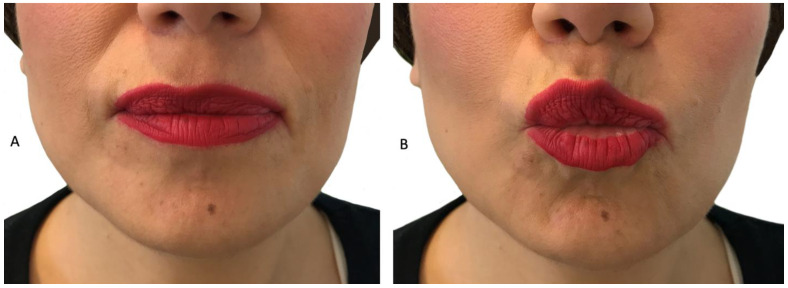
Patient P2: a female FSHD patient, 38 years old with late onset of the disease presenting with weakness of the left perioral muscle. The patient has the inability to whistle or drink with a straw. (**A**): patient with lips relaxed (**B**): patient trying to whistle.

Then, the triad of facial mimic muscles innervated by the facial nerve, were clinically evaluated. The degree of mimic muscle weakness and severity was assessed for the frontalis, the perioral and the periocular muscles (Figure 3). For the evaluation of muscle weakness, which is rather empirical due to the absence of a scale, the criteria set by Kazakov in 1969 and revised in 1993 were implemented [29]. These criteria classify muscle weakness as pre-symptomatic, mild, moderate and severe as follows:

Pre-symptomatic: There were no complaints regarding motor disturbances by the patients, whereas mild muscle weakness and atrophy was observed after specialized tests.

Mild: Complaints of motor disturbances were observed only while performing specific motor acts (such as performing tasks with lifted arms or walking and running on rough surfaces). Upon clinical examination, mild and moderate atrophy (no more than 50% of muscle wasting and reduced size) was reported, a slight weakness of orbital parts (for instance, when the patients attempt to forcefully close their eyes, their eyelashes are not fully absorbed in the depth of the eyelids, they also have weak or absent radial wrinkles); and a slight weakness of the orbicularis oris (as when the patients attempt to whistle asymmetry of the lips or the mouth occurs).

Moderate: Complaints of motor disturbances were reported while performing definite motor acts (difficulties in lifting arms above shoulder level, in walking and in running due to one or both feet hanging down). Upon clinical examination, moderate or severe muscle atrophy was observed (muscle wasting was over 50% compared to usual size), severe weakness of orbital parts (while attempting to close the eyes, the eyelids just touch, along with a complete absence of radial wrinkles), and severe weakness of the orbicularis oris (the patient is unable to whistle or to puff his cheeks).

Severe: Inability of the patient to raise his arms above horizontal level or extend his feet. On clinical examination severe atrophy or disappearance of isolated muscles was observed, loss of orbital and palpebral part of the orbicularis oculi muscles (the patient was unable to close the eyes and sclera were visible), and severe loss of function of the orbicularis oris muscles (patient was unable to stretch out their lips).

In this clinical scale, the degree of severity ranged from 0–5, with 0–1.25 being characterized as pre-symptomatic, 1.26–2.50 as mild, 2.51–3.75 as moderate and 3.76–5.00 as severe. According to the degree of severity, each patient received a single digit number score from 1 to 5. Then, DNA fragment of FSHD1 locus patients was correlated to clinical manifestations as far as the aforementioned orofacial muscles were concerned.

For the statistical analysis, we determined the time of first examination as “point zero” instead of using the uncertain reported age of onset of symptoms. The time at which the patient had the initial symptoms is a clinically important milestone, according to several researchers [30]. Statistical analysis was performed in R (R Core Team, 2021). Firstly, we visually examined the association between the DNA fragment size and the degree of muscle weakness (frontalis muscle, periocular muscle, right perioral muscle, and left perioral muscle) through scatterplots. To numerically quantify the strength of the associations, though, we computed Spearman’s correlation coefficients together with the respective *p*-values. Multivariate regression analyses adjusting for age and sex were also performed.

## 3. Results

A contracted EcoRI/AvrII fragment between 7.5 kb–39 kb was revealed after molecular analysis thus confirming the diagnosis for FSHD1. Small sizes (<15 kb) are compatible with infantile sporadic and more severe cases (<15 kb).

Table 1 shows the 52 patients diagnosed with FSHD, the DNA fragment size and the degree of muscle weakness for the frontalis, the perioral and the periocular muscles.

**Figure 4:**
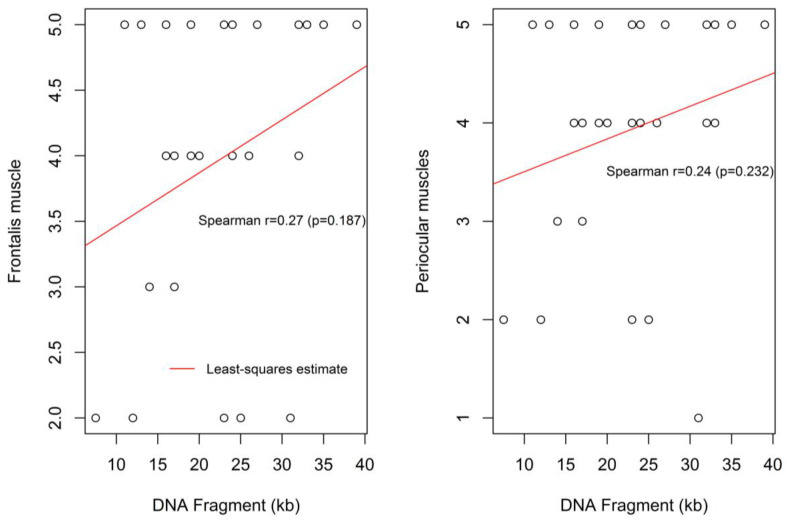
Correlation between the DNA fragment size and the severity of frontalis muscle weakness and the periocular muscle weakness.

There appears to be a positive non-significant correlation between the DNA Fragment (kb) size and frontalis muscle weakness (r = 0.27, *p* = 0.187), and periocular muscle weakness (r = 0.24, *p* = 0.232) (Figure 4). Specifically, with regards to the frontalis muscle 25 out of 52 patients expressed impairment in controlling forehead wrinkling. Of those 25 patients, 18 patients presented with severe weakness of forehead wrinkling, two patients with moderate weakness and five patients with a mild degree of weakness.

Weakness in contraction of the periocular muscles was found in 27 out of 52 patients. Of them, 20 patients presented with severe weakness accompanied with bilateral “signe de cils”; two patients had moderate weakness in closing their eyelids, four patients presented with a mild degree of weakness and one patient revealed a pre-symptomatic degree of weakness. Figure 4 shows the moderate and non-statistically significant correlation between the DNA fragment size and the severity of the periocular (P) muscle weakness.

**Figure 5:**
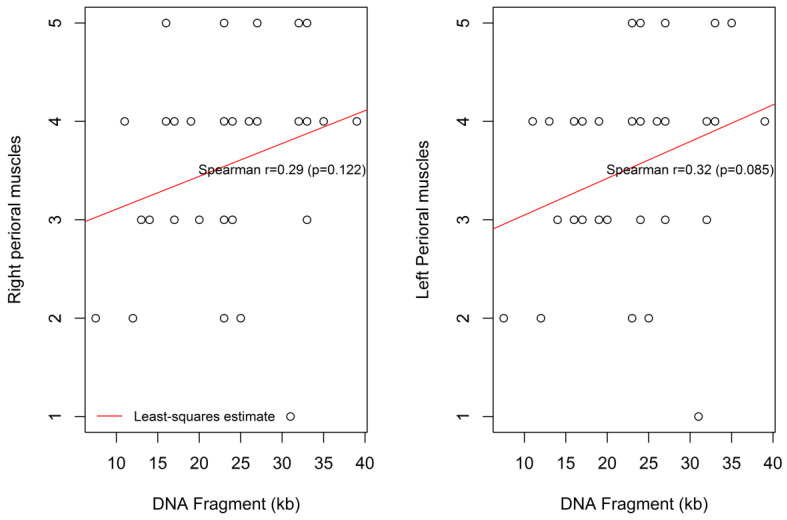
Correlation between the DNA fragment size and the degree of the left and the right perioral muscle weakness.

Similar, but marginally non-significant, trends were found for the association between the DNA Fragment (kb) size and the degree of the left and right perioral muscle weaknesses, (r = 0.29, *p* = 0.122) and (r = 0.32, *p* = 0.085), respectively (Figure 5).

Upon the perioral muscle evaluation, 30 out of 52 patients exhibited weakness in contraction of the lips. Twenty-nine patients presented with right perioral muscular weakness and all 30 patients showed weakness of the left perioral muscle. Regarding the right perioral muscle group, 17 patients presented with severe weakness; seven patients had moderate weakness, four patients had a mild degree of weakness and one patient presented with a pre-symptomatic degree of weakness. Furthermore, concerning the left perioral muscle group 17 patients presented with severe weakness; eight patients had moderate weakness; four patients presented with a mild degree of muscular weakness and one patient exhibited a pre-symptomatic degree of weakness.

Multivariate analysis yielded similar results (Table 2), showing a positive but non-significant correlation between the DNA fragment size and the degree of muscle weakness in all cases. Thus, the possible associations seen through Spearman’s correlation coefficients were not due to age or sex. For example, one-unit increase in the DNA Fragment (kb) size was associated with 0.04 (95% CI: −0.01–0.09) increase in the left perioral muscle weakness score (*p* = 0.120), independently of age at examination and sex. Furthermore, age and sex did not affect the degree of muscle weakness.

Still, clinically, it was noted that 20 out of 52 patients presented with bilateral asymmetry of the perioral muscles. Specifically, 10 of those 20 patients revealed a greater involvement of the right side of the lower facial nerve while the other 10 patients had involvement of the left side. In addition, eight out of the 52 patients exhibited a reduction of muscle activity in the right nasolabial fold, while six out of the 52 had a reduction in the left nasolabial fold (data not shown).

## 4. Discussion

In dentofacial orthopedics, the concept of muscle forces affecting facial morphology, and leading to a wide spectrum of malocclusion is not new [31]. Many researchers have reported that muscular activity, has an effect on the facial skeleton [32]. Mechanical forces exerted by the facial musculature on the underlying skeleton, contribute to the homeostasis of the bones and during the course of dentofacial orthopedic treatment result in various changes in their size and shape. Therefore, any genetic variation that has an impact on muscular composition and subsequently on the mechanical properties would also affect the skeletal configuration especially during the active phase of growth and development [33].

The role of muscular disequilibrium on the development of dentofacial malocclusion has been quite thoroughly investigated at both a histological and cellular level. Recently conducted studies explored the association between genetic variants involved in muscular activity and deviations of the skeletal and facial characteristics [34].

Genotype–phenotype association studies do provide important knowledge that could possibly translate into preventative measures, prognostic value, better clinical outcomes, and potentially more rewarding clinical practice. Clinical orthodontics and dentofacial orthopedics aim at balance, harmony and symmetry in skeletal growth thus, providing a good foundation for teeth to be aligned into an ideal occlusion. Ideal size and position of the facial skeleton in all three planes of space also contributes to enhanced facial esthetics. Therefore, any knowledge of the genetic determinants of these dimensions will contribute greatly to the understanding of craniofacial growth and development [35]. Human genes are found to be responsible for deviations in skeletal, dental, and soft tissue features in growing patients. The early identification of such etiologic factors, would enable the clinician to achieve appropriate diagnosis and treatment outcomes, contributing to the overall enhancement of public health [36].

In the present study, the correlation between the D4Z4 fragment size and the involvement of the perioral, periocular and frontalis muscles was found to be weak to moderate and non-significant or marginally non-significant (left perioral muscle). Our findings are in agreement with those of Mul et al. who reported that the strength of lip compression was not decreased in comparison with the healthy control group even though the orbicularis oris muscle controls the lip movement [37]. This fact could suggest that lip and mouth weakness is affected by a variety of factors apart from the orbicularis oris impairment [37]. Such causes might be other facial muscles, like the buccinator or risorius muscles. Decreased cheek compression strength of FSHD patients could encourage the hypothesis of buccinator involvement, but further research is necessary in order to study the exact associations of other facial muscle groups.

Facial weakness in some cases may appear very early and become unnoticed by the patients themselves. Often, it is considered a sub-clinical finding that is reported after a clinical consultation. In the present group of FSHD patients, a substantial number of patients exhibited inability or weakness in whistling and closing their eyelids. However, cases with facial manifestations before the age of 5 years are defined as an infantile type of FSHD according to Brouwer et al. [38].

According to Padberg, before the establishment of genetic testing and relying solely on clinical signs and examination, absence of facial weakness was reported in only two out of 113 patients [39,40]. However, FSHD is a progressive disease and its duration can influence the severity of clinical symptoms and the subsequent evaluation.

Tawil ascertained Padberg’s findings, suggesting that in the absence of facial weakness, diagnosis of FSHD is acceptable only if the majority of the family members present impairment of the mimic musculature [41]. Moreover, it was reported that no atrophy existed in the tongue muscles of the patients examined. Shimizu et al. reported a patient who was possibly associated with a congenital form of FSHD that was accompanied with hearing impairment and tongue atrophy [42]. Since the previously mentioned study took place prior to the establishment of molecular genetic testing, their diagnosis was not confirmed genetically [42]. In a series of 151 Japanese patients with FSHD, 4.6% of them exhibited tongue atrophy that was confirmed by findings in both electromyography and magnetic resonance imaging. Those patients reported an early onset of the disease and possessed a considerable genetic defect [43].

According to Mul et al., 30% of the variance involving facial muscle function was strongly correlated to D4Z4 repeat size [44]. Increased facial weakness was only demonstrated in patients with less than 5 D4Z4 repeat units, which concords with the majority of the findings in our study. The factors that lead to the increased clinical variability in FSHD are currently under investigation. Mul et al. report that patients with repeat array sizes between five to nine units correspond to only 10% of the variance in the severity of the disease. It is worth mentioning that the currently unknown factors which contribute to the variability of the disease may include a fusion of epigenetic, organismal, lifestyle and environmental elements [44].

It has been expressed by numerous researchers, that the form of muscular involvement in FSHD is determined by genetic and epigenetic factors. Whether this involvement is different according to the muscle group affected has been thoroughly investigated. The D4Z4 repeat array size has a stronger impact on the amount of facial weakness compared to upper and lower extremity impairment. This conclusion is confirmed by a variety of studies, stating that patients with no involvement of the facial musculature, have in general repeat array sizes of more than 30 kb (relatively 7 units) [43,45,46].

Such results may raise questions epitomizing the fact that facial musculature impairment, as an initial symptom of FSHD, is closely correlated to changes in DUX4 expression levels more than different muscle groups. Although there is a lack of current literature regarding facial muscles in FSHD, recent studies propose an interrelationship among DUX4 and myogenic Pax3 and Pax7 homedomain transcription factors. The selective relationship with the previously mentioned factors and not with others such as Pitx2 and Tbx1 may lead to the speculation that facial muscles are more vulnerable to DUX4 damage during growth [47].

Recently, Loonen et al., evaluated facial weakness in 87 patients with FSHD and they concluded that approximately 10% of the patients had very mild weakness [48]. Although many studies correlated a very mild FSHD phenotype, possibly without facial involvement and with small deletions (large EcorI/AvrII fragments), this study expands the heterogeneity of these findings evaluating the orofacial muscle weakening with the range of contracted alleles in FSHD1 genetic locus. It is evident in our study that patients with small DNA fragment sizes could present a highly rated degree of facial muscle weakness.

However, the epigenetic underlying etiology in FSHD seems to explain the vast intra- and inter-family phenotypic heterogeneity even in patients with the same number of D4Z4 repeats on contracted 4qA haplotype [49].

According to Ruggiero et al. and Ricci et al., carriers of small D4Z4 alleles present various phenotypic manifestations of the disease, often with facial weakness being absent in some subjects [4,6]. The documentation of the increasing amount of such cases in large cohort studies conducted recently, indicate that there might be additional factors (such as the genetic background) contributing to the phenotypic expression differences of FSHD, even in subjects which carry the same molecular signature [4,6].

In agreement with the aforementioned studies the present investigation demonstrated a phenotypic variability of the orofacial musculature examined, which is only weakly correlated to the D4Z4 allele contraction in a group of patients genetically diagnosed with FSHD. However, the association was similar (both in magnitude and direction) for all muscles. Similar trends were also seen, after adjusting for sex and age.

Although several studies have proposed a roughly inversed correlation between the D4Z4 repeat size and the clinical severity, it is worthwhile mentioning that recently a phenotypic variability has been reported in the literature among subjects carrying repeat arrays of similar or even of the same size [4]. Molecular testing, measurement of D4Z4 alleles size, 4qA polymorphism, as well as D4z4 methylation status have been fundamental criteria for the diagnosis and management of FSHD subjects [50]. However, clinical manifestations of the disease should be seriously taken into consideration in the process of diagnosis. According to Ruggiero et al., for carriers with decreased repeat arrays molecular testing proved to be inadequate for diagnosis. Thus, orofacial muscle weakening and its association with a D4Z4 contraction alone may not have the significance to serve as a prognostic biomarker, due to the weak to moderate association also highlighted in the present study.

Furthermore, the impairment of the facial muscle groups that were examined in our study (frontalis, periocular and orbicularis oris muscles) may impact the patient’s extent of facial expression, thus hindering social communication aspects. Moreover, the psychological outcome of facial weakness has not yet been investigated, although it could have a major impact.

Often, patients with neuromuscular disorders are unaware of their condition, although the craniofacial manifestations of FSHD precede the systemic ones, especially at young ages. Health care providers involved like orthodontists, dentists and pediatricians should be able to recognize the clinical manifestations of FSHD and guide their patients to the specialist and to obtain appropriate genetic testing. Still, the meticulous assessment of muscular weakness is recommended prior to orthodontic treatment planning due to muscular dystrophies progressive nature, which could worsen treatment prognosis.

The further elucidation of the genetic involvement in the growth and function of the dentofacial complex and musculature will contribute to better patient care aimed at enhanced function, facial aesthetics and well-being. Finally, monitoring the correlation between D4Z4 fragment size and the severity of orofacial muscle weakness in prospective longitudinal observational studies of subjects diagnosed with FSHD, even by implementing contemporary imaging techniques such as magnetic resonance (MRI), could provide essential information regarding the onset, progression and the manifestations of FSHD in the orofacial complex [51].

## 5. Limitations

The limitations of this study can be primarily attributed to the sample size. Still, FSHD patients comprise a rare disease and the collection of large samples can be challenging. Additionally, all patients were of Caucasian origin and this constitutes another limitation. Further investigations should examine different ethnic groups and obtain larger sample sizes. The evaluation of the muscle weakness was also empirical. The employment of continuous data consisting of measurements recorded on an objective scale instead of the empirical assessment would contribute to the reliability and repeatability of the results.

## 6. Conclusions

- In FSHD patients, the molecular genetic testing depicted altered DNA fragments sized from 7.5 to 39 kb with a mean of 23.89 kb;
- Fragment size provided a weak to moderate correlation with the severity of muscle weakness in the muscular triad (frontalis, periocular, right and left perioral muscles) that was examined;
- Orofacial muscle weakening and its association with a D4Z4 contraction alone may not have the significance to serve as a prognostic biomarker, due to the weak to moderate association;
- Further investigation is needed to clarify the degree of genetic involvement in the growth and development of the facial musculature and the dentofacial complex in FSHD patients.

## Figures and Tables

**Table 1:** DNA fragment size and degree of muscle weakness assessed for the frontalis, the perioral and the periocular muscles in the 52 patients diagnosed with FSHD.

Patient	Gender	Age	DNA Fragment (kb)	Frontalis Muscle	Periocular Muscles	Perioral Muscles Right	Perioral Muscles Left
1.	M	64	24	-	-	3	3
2.	M	34	24	-	4	-	-
3.	M	39	24	5	-	4	-
4.	M	72	24	-	5	-	5
5.	F	13	-	-	-	-	-
6.	F	13	-	-	-	-	-
7.	M	33	23	-	-	5	-
8.	F	42	-	-	-	-	-
9.	F	23	23	-	2	3	-
10.	F	36	23	-	5	-	-
11.	M	70	-	-	-	-	-
12.	F	28	13	5	5	3	4
13.	F	30	35	5	5	4	5
14.	M	52	17	3	-	-	-
15.	F	26	17	-	3	-	4
16.	M	62	23	-	4	-	4
17.	F	30	23	5	-	4	5
18.	M	55	32	5	-	5	-
19.	M	54	32	-	5	-	-
20.	F	48	16	5	-	5	4
21.	F	20	16	4	4	4	3
22.	F	51	33	-	-	5	-
23.	F	50	33	-	5	4	-
24.	M	55	27	5	-	-	3
25.	F	50	27	-	-	-	5
26.	M	28	27	-	-	4	-
27.	F	36	33	-	4	3	4
28.	M	20	33	-	-	-	5
29.	M	71	17	-	4	3	3
30.	F	33	17	4	-	-	-
31.	M	41	17	-	-	4	-
32.	F	66	19	-	4	4	3
33.	F	66	32	4	-	-	3
34.	F	37	32	-	-	4	-
35.	F	42	32	-	-	-	4
36.	M	71	20	4	4	3	3
37.	F	82	31	2	1	1	1
38.	M	49	25	2	2	2	2
39.	M	36	14	3	3	3	3
40.	M	54	32	-	4	-	-
41.	M	23	33	5	-	-	-
42.	M	46	26	4	4	4	4
43.	M	21	23	2	-	2	2
44.	M	18	7.5	2	2	2	2
45.	F	30	12	2	2	2	2
46.	F	36	11	5	5	4	4
47.	F	18	39	5	5	4	4
48.	F	36	19	4	-	-	-
49.	F	42	24	4	-	-	4
50.	F	62	27	-	5	5	4
51.	M	36	16	-	5	-	-
52.	M	25	19	5	5	-	4

**Table 2:** Multivariate analysis for the association between DNA Fragment (kb) and frontalis muscle weakness, periocular muscle weakness, right perioral muscle weakness, and left perioral muscles weakness, adjusting for gender and age.

Frontalis Muscle		Coef.	95% C.I.	*p*-Value
DNA Fragment (kb)	per unit	0.05	(−0.01, 0.11)	0.120
Gender	F	-	-	-
	M	−0.38	(−1.34, 0.59)	0.426
Age at first examination (years)	per unit	−0.01	(−0.04, 0.02)	0.347
Intercept		3.63	(1.95, 5.31)	<0.001
Periocular muscles				
DNA Fragment (kb)	per unit	0.04	(−0.03, 0.10)	0.237
Gender	F	-	-	-
	M	0.14	(−0.91, 1.19)	0.786
Age at first examination (years)	per unit	−0.01	(−0.04, 0.02)	0.651
Intercept		3.28	(1.51, 5.04)	0.001
Right perioral muscles				
DNA Fragment (kb)	per unit	0.03	(−0.02, 0.09)	0.225
Gender	F	-	-	-
	M	−0.19	(−1.04, 0.66)	0.655
Age at first examination (years)	per unit	−0.00	(−0.03, 0.02)	0.776
Intercept		3.03	(1.43, 4.63)	0.001
Left perioral muscles				
DNA Fragment (kb)	per unit	0.04	(−0.01, 0.09)	0.120
Gender	F	-	-	-
	M	−0.25	(−1.04, 0.54)	0.519
Age at first examination (years)	per unit	−0.01	(−0.03, 0.01)	0.240
Intercept		3.27	(1.81, 4.72)	<0.001

## Data Availability

The data presented in this study are available on request from the corresponding author. The data are not publicly available due to GDPR policy.
